# P048 When treating to prevent blindness causes harm: balancing the risks in giant cell arteritis

**DOI:** 10.1093/rap/rkag113.078

**Published:** 2026-11-03

**Authors:** Fiona Starkie

**Affiliations:** Gloucester Royal Hospital, Gloucester, United Kingdom

## Abstract

**Introduction:**

Giant cell arteritis (GCA) is a medical emergency because delayed treatment may result in irreversible visual loss. Consequently, high-dose glucocorticoids are frequently commenced before diagnostic confirmation. However, corticosteroids carry substantial risks, particularly in older adults with multimorbidity, where treatment-related complications may rival or exceed the consequences of disease itself. This case illustrates the challenges of managing suspected GCA in a patient with low inflammatory markers, who subsequently developed profound steroid-induced psychosis. The case highlights the importance of multidisciplinary decision-making, continual reassessment of diagnosis, and balancing the competing risks of undertreated vasculitis against glucocorticoid-related harm.

**Case description:**

A gentleman in his seventies was referred via the same-day emergency care after four months of left-sided temporal headache and jaw discomfort. Although his inflammatory markers were normal (ESR 12 mm/hr), temporal artery ultrasound demonstrated a 2 cm halo sign affecting the proximal left frontal branch, and he commenced on high-dose prednisolone (60 mg daily) for presumed GCA prior to specialist review.

At rheumatology assessment three weeks later, he denied symptoms of polymyalgia rheumatica, visual disturbance or limb claudication. Examination demonstrated persistent left-sided scalp discomfort with preserved temporal artery pulsation and no visual or peripheral vascular abnormalities. Extensive Bateman purpura were present and felt due to combination treatment with steroids and blood thinners. He complained of insomnia, urinary hesitancy and disorientation since commencement of steroid therapy.

Within days, he experienced behavioural change characterised by agitation, grandiosity, religious delusions, insomnia and disinhibited behaviour. Adherence to treatment was variable and his mental state deteriorated further, requiring police detention before admission under the Mental Health Act.

During admission, the patient developed recurrent episodes of supraventricular tachycardia and atrial flutter, alongside chest pain and haemoptysis. CXR demonstrated focal consolidation, raising concern for MRSA pneumonia. Ongoing confusion and persistent headache prompted investigation for alternative neurological causes. CT Head was unremarkable, while lumbar puncture demonstrated only mildly elevated cerebrospinal fluid protein. Viral encephalitis remained a differential diagnosis, and empirical antiviral treatment was commenced following microbiology advice.

Given the ongoing concerns regarding steroid-induced psychosis and immunosuppression, prednisolone was rapidly tapered from 60 mg to 30 mg before recommencing a routine tapering schedule. Additional immunosuppression was considered but deferred because of infection risk, uncertain benefit and concerns regarding treatment adherence. Risperidone was commenced by psychiatry to manage steroid-induced mania with psychotic features. The patient is still undergoing treatment as an inpatient at the local psychiatric hospital.

**Discussion:**

This case illustrates the complexity in suspected GCA, where the imperative to prevent irreversible visual loss must be balanced against the harms of empirical high-dose glucocorticoid therapy.

Although temporal artery ultrasound demonstrated a unilateral halo sign, the patient’s persistently normal inflammatory markers, absence of visual symptoms and subsequent clinical course reduced confidence that active vasculitis remained the principal driver of headache. In this case, a temporal artery biopsy was not done. Nevertheless, the catastrophic consequence of undertreated GCA made early withdrawal of corticosteroids difficult.

The principal challenge in this case was balancing the risk of undertreating possible GCA against the substantial harm caused by glucocorticoid therapy. The co-existence of pneumonia, suspected viral encephalitis and pre-existing cognitive impairment complicated diagnostic interpretation and increased the patient’s vulnerability to delirium. Although the neuropsychiatric presentation was most consistent with corticosteroid-induced psychosis, alternative diagnoses such as viral encephalitis, delirium secondary to sepsis and cerebral vasculitis were considered.

Decision-making was further influenced by poor patient insight and fluctuating treatment adherence, complicating the implementation of treatment plans. Previous longstanding mild cognitive impairment, which wasn’t initially appreciated, may have lowered the threshold for neuropsychiatric complications.

This case illustrates the value of multidisciplinary care. Rheumatology, psychiatry, neurology, microbiology and acute medicine teams collaborated to reassess the diagnosis, investigate and determine the safest treatment strategy for the patient. Ultimately, management departed from conventional GCA pathways, with accelerated steroid tapering. GCA management extends beyond preventing visual loss. Individual patient characteristics, treatment harm and diagnostic uncertainty must all be considered. In selected patients, deviation from standard treatment pathways may represent the safest and most patient-centred approach.

In this case, profound steroid-induced mania with psychotic features resulted in detention under the Mental Health Act, prolonged admission and substantial morbidity.

**Key learning points:**

1. High-dose glucocorticoids can unmask underlying psychiatric disorders and can result in profound morbidity, especially in older adults.

2. Management of GCA involves balancing competing risks rather than focusing exclusively on disease control.

3. Normal inflammatory markers do not exclude GCA, and ultimately diagnosis is based on a combination of clinical assessment and investigation.

4. Multi-disciplinary collaboration is essential in complex cases to support diagnostic reassessment, management of complications and individualised treatment decisions when standard GCA pathways are no longer appropriate.

5. Suspected GCA requires urgent treatment, but diagnostic uncertainty should be revisited continually. The diagnosis should be reassessed as new clinical information emerges, particularly when features are atypical or significant treatment side effects occur.

6. In selected patients, deviation from standard treatment pathways may represent the safest and most patient-centred approach.

